# Use of smartphone-based instant messaging services in medical practice: a cross-sectional study

**DOI:** 10.1590/1516-3180.2020.0010.R1.28032020

**Published:** 2020-04-22

**Authors:** Israel Junior Borges do Nascimento, João Antonio de Queiroz Oliveira, Iago Souza Wolff, Laura Defensor Ribeiro, Maíra Viana Rego Souza e Silva, Clareci Silva Cardoso, Maurice Mars, Antonio Luiz Ribeiro, Milena Soriano Marcolino

**Affiliations:** I PharmB. Medical Research Specialist, Medical School and TeleHealth Center, University Hospital, Universidade Federal de Minas Gerais (UFMG), Belo Horizonte (MG), Brazil; and Medical Research Specialist, Medical College of Wisconsin, Milwaukee, Wisconsin, United States.; II PharmD. MSc. Pharmacist, Medical School and TeleHealth Center, University Hospital, Universidade Federal de Minas Gerais (UFMG), Belo Horizonte (MG), Brazil; III MD. Physician, Medical School and TeleHealth Center, University Hospital, Universidade Federal de Minas Gerais (UFMG), Belo Horizonte (MG), Brazil.; IV MD. Physician, Medical School and TeleHealth Center, University Hospital, Universidade Federal de Minas Gerais (UFMG), Belo Horizonte (MG), Brazil.; V MD. Physician, Medical School and TeleHealth Center, University Hospital, Universidade Federal de Minas Gerais (UFMG), Belo Horizonte (MG), Brazil.; VI MD, MSc, PhD. Professor, Department of Public Health, Medical School and TeleHealth Center, Universidade Federal de São João del-Rei, Divinópolis, Brazil.; VII MBChB, MD. Professor, Department of TeleHealth, Nelson R. Mandela School of Medicine, University of KwaZulu-Natal, Durban, South Africa.; VIII MD, PhD. Professor, Medical School and TeleHealth Center, University Hospital, Universidade Federal de Minas Gerais (UFMG), Belo Horizonte (MG), Brazil.; IX MD, MSc, PhD. Professor, Medical School and TeleHealth Center, University Hospital, Universidade Federal de Minas Gerais (UFMG), Belo Horizonte (MG), Brazil.

**Keywords:** Telemedicine, Mobile applications, Smartphone, Evidence-based Medicine, Jurisprudence, Medical legislation, Instant messaging applications, Clinical practice

## Abstract

**BACKGROUND:**

Instant messaging services (IMS) are widely used in medical practice.

**OBJECTIVE:**

To evaluate perceptions regarding use and usability of IMS within clinical practice and assess users’ knowledge of the ethical and legal context involved in using IMS within medical practice.

**DESIGN AND SETTING:**

Cross-sectional study conducted in different hospitals and medical institutions in Minas Gerais, Brazil.

**METHODS:**

Medical students, medical residents, primary care physicians and specialist doctors answered an online questionnaire regarding epidemiological data, graduation level and use of IMS for medical communication. Responses were collected over a five-month period and data were assessed using the IBM-SPSS software.

**RESULTS:**

484 people answered the questionnaire: 97.0% declared that they were using IMS for medical-related purposes; 42.0%, to elucidate medical concerns every week; 75.0%, to share imaging or laboratory tests and patients’ medical records; and 90.5%, to participate in clinical case-study private groups. Moreover, only 37.0% declared that they had knowledge of the legislative aspects of use of smartphones within clinical practice. Differences in the frequency of discussion of medical concerns within the daily routine between student/residents and general practitioners/specialists, and in the frequency of image-sharing and patient-guiding/assistance between students and medical doctors, were observed.

**CONCLUSIONS:**

Our results provide reliable proof that medical doctors and students use IMS, as a tool for clinical case discussions, interactions between healthcare providers and patients, or dissemination of knowledge and information. Nonetheless, because of limitations to the ethical and legal regulations, evidence-based discussions between authorities, academics and medical institutions are needed in order to fully achieve positive outcomes from such platforms.

## INTRODUCTION

Use of telemedicine has become more frequent and more convenient for addressing medical issues.^1^ It is perfectly suited for use in the modern world because of its cost-efficiency, its availability in remote and rural areas, the improved access to care that it provides and its shorter response time. It can also improve the consistency and quality of healthcare.^2^

In 2018, data from the International Telecommunications Union showed that there were more than 3.9 billion active mobile phone (cellphone) subscriptions worldwide.^3^ More than 165,000 health-related applications (apps) have been designed and 62% of smartphone holders use their phone to obtain health advice.^4^^,^^5^

Social media and smartphone-based instant messaging services (IMS) have exploded in popularity over recent years. Instant messaging services, such as WhatsApp and iMessage, have become a very common way to communicate, for personal and professional purposes.^6^ The use of these services has become progressively more popular within the field of medicine, and they serve to connect doctors to patients, to other doctors and to other healthcare professionals.^7^ This paradigm shift in medicine, created through popular communication applications, is of relevance both to developed and developing countries because of the economic, political and social issues that arise through use of these means of communication. 

Within the context of telemedicine programs, it is important to consider the legal perspective relating to contact between healthcare professionals and their patients or between these professionals and their colleagues. There is a need to avoid problems relating to privacy issues and medical malpractice, as well as to avoid fraud and abuse. 

In Brazil, the Federal Medical Council (Conselho Federal de Medicina, CFM) recently raised concerns regarding indiscriminate use of these apps among healthcare professionals and their patients.^8^ In April 2017, the CFM published a memorandum (reference number 14/2017) regulating the use of IMS for physician-physician communication and for patient care. This policy states that these apps must not replace face-to-face consultations: they should be a complement to regular medical practice. More recently, the promulgation of a data protection law in Brazil, to regulate the use of personal information by third parties, has also given rise to more discussion on the use of data for medical purposes.^9^ It is notable that sensitive personal information is commonly exposed, whether in social media, on television channels or between companies.^10^^,^^11^ This is symptomatic of the current lack of legal information and boundaries for medical doctors with regard to information sharing. No research so far has analyzed such unawareness among health professionals. 

## OBJECTIVE

To ascertain perceptions regarding use and usability of these apps within clinical practice and to assess users’ levels of knowledge about the ethical and legal context involved in use of these apps within medical practice.

## METHODS

This was a cross-sectional study conducted in the state of Minas Gerais, in southeastern Brazil. The inclusion criteria were that the subjects needed to be any of the following: (1) medical students at a public university in Belo Horizonte, the state’s capital and largest city, with 2.5 million inhabitants; (2) medical residents at this university’s teaching hospital; (3) primary care physicians registered in the database of the Telehealth Network of Minas Gerais (TNMG), which is a public Telehealth service that was providing primary care services for 814 municipalities in Minas Gerais at that time; or (4) specialist doctors at the university’s teaching hospital or registered in the database of the TNMG. 

A standardized questionnaire containing 10 closed-ended questions and 4 open-ended questions was developed by an interdisciplinary group of specialists. It was then hosted in a survey administration application (Google Forms). In total, 6591 e-mails were sent out containing an invitation to participate, a description of the research and an access link to the questionnaire. 

The questions addressed the following: age; professional experience; frequency of instant messaging service use for medical-related purposes (in days); participation in health-related app groups (number of groups involved); use of instant messaging apps for clinical-support tools (yes/no), or for patient follow-up care or monitoring; and perception of benefits from these apps for enabling clinical solutions (using a defined scale). In addition, one question investigated the subjects’ level of knowledge of the legal aspects of sharing and discussing medical matters using instant messaging apps. An invitation to participate in the survey was sent out electronically to eligible respondents, who had been identified through the academic office of the medical school, the residency program office of the university hospital and the TNMG.

All the data were assessed using the IBM Statistical Package for the Social Sciences (SPSS) for Windows, version 19.0. Categorical variables were presented as absolute numbers and relative frequencies and continuous variables as medians and interquartile ranges, since the distribution was not normal. The participants were categorized according to their level of education as medical students, medical residents, medical specialists and general physicians. The Kolmogorov-Smirnov test and Kruskal-Wallis test were used to assess differences among groups. We decided to compare two main clusters of participants: medical students and medical residents versus specialists and general practitioners. This was because of the possible correlations and similarities of patterns among these individuals (whether still graduating or professionally restricted). 

This investigation was approved by the local Research Ethics Committee, through protocol number 82097018.0.000.5149, and consent was obtained from all participants in accordance with the Helsinki Declaration. 

## RESULTS

The total study population consisted of 484 people. The median age was 27 years (interquartile range, IQR 23-33), and most of the participants were 20-30 years of age (60.0%). With regard to the respondents’ educational level, 41.0% were medical students, 22.0% resident physicians, 21.0% specialist doctors and 16.0% general doctors. Overall, among the medical students (n = 197), 36.0% were in the fifth or sixth year of medical school, 32.5% in the third or fourth year and 31.5% in the first or second year. Among the physicians, a considerable proportion of the participants stated that they had between 0 and 5 years of experience (33.0%).

Table 1 shows details of the subjects’ responses relating to usage of instant messaging services, according to educational status. Most respondents (97.0%) declared that they were using these apps for medical-related purposes, with higher prevalence among residents/students than among general practitioners/specialist s(298 versus 163; P ≤ 0.001). There was a significant difference between medical students/residents and general practitioners/specialist regarding frequency of use for medical purposes, except for irregular daily use. The participants’ frequency of use of instant messaging apps for medical purposes was categorized as a few times a day or multiple daily access. Additionally, 42.0% of the participants reported that they were using instant messaging services every week to elucidate medical concerns, in the form of either professional-to-professional interactions or group settings, with higher weekly prevalence among medical student/residents than among specialists/general practitioners (41.9% versus 12.6%; P = 0.012 respectively). Most of the respondents perceived advantages from using these apps in clinical practice (55.2%), while 1.4% of those who had already used instant messaging apps considered that they did not help whatsoever within clinical practice (i.e. they believed that these apps did not provide any reasonable benefits).

**Table 1.** Online questionnaire responses according to multiple categories

	Total (n = 474)	Medical student (n = 197)	Medical resident (n = 103)	Medical specialist (n = 98)	General practitioner (n = 76)	Student/resident versus general physician/specialist (P-value)
Use of instant medical communication app	461 (97.2)	195 (99.0)	103 (100.0)	90 (91.8)	73 (96.1)	≤ 0.001
Use of WhatsApp	460 (97.0)	195 (99.0)	103 (100.0)	90 (91.8)	72 (94.7)	≤ 0.001
Use of Facebook Messenger	272 (57.3)	132 (67.0)	58 (56.3)	51 (52.0)	31 (40.8)	≤ 0.001
Use of Skype	53 (11.1)	22 (11.0)	8 (1.0)	15 (15.0)	8 (10.0)	0.283
Use of Telegram	66 (13.9)	25 (12.0)	16 (15.0)	15 (15.0)	10 (13.0)	0.831
Use of iMessage	39 (8.2)	15 (7.0)	9 (9.0)	9 (9.0)	6 (8.0)	0.812
Use of Viber	15 (3.1)	1 (0.01)	8 (8.0)	5 (5.0)	1 (1.0)	0.788
Use of Hangouts	14 (2.9)	9 (4.0)	1 (1.0)	4 (4.0)	0 (0.0)	0.521
Frequency of use for medical purposes						
No use	15 (3.2)	2 (1.0)	0 (0.0)	10 (10.2)	3 (3.9)	≤ 0.001
Rare	44 (9.2)	8 (4.1)	3 (2.9)	17 (17.3)	16 (21.1)	≤ 0.001
A few times a day	177 (37.4)	78 (39.6)	43 (41.7)	25 (25.5)	31 (40.8)	0.077
Multiple daily access	238 (50.2)	109 (55.3)	57 (55.3)	46 (46.9)	26 (34.2)	0.003
Number of discussion groups involved						
No group	45 (9.3)	13 (6.6)	1 (1.0)	22 (22.4)	9 (11.8)	≤ 0.001
1-2 groups	100 (20.7)	25 (12.7)	11 (10.7)	36 (36.7)	28 (36.8)	≤ 0.001
3-5 groups	182 (37.6)	87 (44.2)	46 (44.7)	19 (19.4)	30 (39.5)	≤ 0.001
More than 5 groups	147 (30.4)	72 (36.5)	45 (43.7)	21 (21.4)	9 (11.8)	≤ 0.001
Frequency of online discussion of clinical cases						
No discussion	42 (8.7)	20 (10.2)	1 (1.0)	16 (16.3)	5 (6.6)	0.061
Rarely	80 (16.5)	28 (14.2)	12 (11.7)	23 (23.5)	17 (22.4)	0.007
Daily	90 (18.6)	31 (15.7)	28 (27.2)	23 (23.5)	8 (10.5)	0.620
Weekly	199 (41.1)	87 (44.2)	52 (50.5)	22 (22.4)	38 (50.0)	0.012
Monthly	63 (13.0)	31 (15.7)	10 (9.7)	14 (14.3)	8 (10.5)	0.752
Perception of use						
Never used	43 (8.9)	20 (10.2)	2 (1.9)	16 (16.3)	5 (6.6)	0.653
Never helped	7 (1.4)	3 (1.5)	2 (1.9)	1 (1.0)	1 (1.3)	0.084
Used but could solve the case without the application	157 (32.4)	77 (39.1)	29 (28.2)	30 (30.6)	21 (27.6)	0.179
Used and considered essential	267 (55.2)	97 (49.2)	70 (68.0)	51 (52.0)	49 (64.5)	0.703
Use for image-sharing purposes						
No use	115 (23.8)	71 (36.0)	6 (5.8)	22 (22.4)	16 (21.1)	0.349
Rarely	122 (25.2)	50 (25.4)	26 (25.2)	24 (24.5)	22 (28.9)	0.791
Daily	44 (9.1)	12 (6.1)	12 (11.7)	16 (16.3)	4 (5.3)	0.206
Weekly	136 (28.1)	45 (22.8)	45 (43.7)	25 (25.5)	21 (27.6)	0.408
Monthly	57 (11.8)	19 (9.6)	14 (13.6)	11 (11.2)	13 (17.1)	0.367
Frequency of patient-guiding orientation						
No use	234 (51.0)	145 (74.0)	50 (49.0)	27 (27.6)	23 (30.3)	≤ 0.001
Rarely	134 (27.7)	37 (19.0)	35 (34.0)	32 (32.7)	30 (39.5)	0.007
Daily	22 (10.7)	3 (2.0)	1 (1.0)	14 (14.3)	4 (5.3)	≤ 0.001
Weekly	44 (9.2)	5 (2.5)	8 (8.0)	21 (21.4)	10 (13.2)	≤ 0.001
Monthly	26 (5.4)	5 (2.5)	8 (8.0)	4 (4.1)	9 (11.8)	0.148
Legal knowledge						
No legal knowledge	121 (25.5)	59 (29.9)	25 (24.3)	18 (18.4)	19 (25.0)	0.105
No literature-based knowledge	180 (38.0)	82 (41.6)	47 (45.6)	23 (23.5)	28 (36.8)	0.003
Literature-based knowledge	173 (36.5)	56 (28.4)	31 (30.1)	57 (58.2)	29 (38.2)	≤ 0.001
Impact on medical practice						
No impact	17 (3.6)	2 (1.0)	0 (0.0)	11 (11.2)	4 (5.3)	≤ 0.001
Negative impact	6 (1.2)	2 (1.0)	1 (1.0)	2 (2.0)	1 (1.3)	0.497
Either positive or negative	342 (72.2)	162 (82.2)	85 (82.5)	51 (52.0)	44 (57.9)	≤ 0.001
Positive impact	109 (23.0)	31 (15.7)	17 (16.5)	34 (34.7)	27 (35.5)	≤ 0.001

Values shown are n (%) or median (interquartile range).

Regarding perceptions of use, there were no statistically significant differences between medical students/residents and specialists/general practitioners. Most of the respondents also declared that they had already used these apps to sharing imaging examinations, laboratory tests or patients’ medical records (76.2%), with no statistically significant differences between medical students/residents and general practitioners/specialists. Additionally, most of the participants belonged to clinical case-study closed groups (90.5%), and frequently more than three groups. Interestingly, medical students/residents were more likely to participate in more than three discussion groups, while general practitioners/specialists more frequently belonged to one or two discussion groups.

A total of 50.6% of the participants stated that they had previously used smartphone-based instant services for guiding and/or advising patients. Lower daily and weekly use of instant messaging apps for patient-guiding was seen among medical students/residents than among general practitioners/specialists (25.75% versus 74.25%; P ≤ 0.001).

With regard to opinions about the impact of instant messaging apps on clinical practice, 72.2% of the respondents considered that these apps could have either a positive or a negative impact on medical practice, while 22.5% considered that these apps were entirely positive. Medical students/residents were less likely to perceive the positive impact of instant messaging apps than were general practitioners/specialists (44.0 versus 55.9%; P ≤ 0.001).

With regard to knowledge of ethics and legal matters, 74.5% of the participants stated that they had previous knowledge of the legislative aspects of use of these apps, although 38.0% of these participants reported that they had never checked this in the official literature. The remainder of the participants stated that they did not have any previous information relating to legal perspectives (25.5%). Regarding legal knowledge gained from the literature, there was a statistically significant difference between medical students/residents and specialists/general practitioners (18.3% versus 18.1%; P ≤ 0.001).

## DISCUSSION

Studies on the impact of instant messaging apps within medicine are rare and still at an initial stage. In the present study, most of the physicians and medical students reported that they were using instant communication apps for medical-related issues, and WhatsApp and Facebook Messenger were the ones most used. The majority of the respondents reported that they were using instant messaging technologies to participate in discussion groups, for a variety of purposes: imaging sharing (radiological, clinical or laboratory), clinical case discussion, knowledge dissemination and, more rarely or not at all, for patient-guiding. Use of instant messaging apps was mostly perceived and evaluated as useful/essential or useful but not mandatory and the perception of negative impacts on medical practice was remarkably low. Most of the participants had not seen any literature-based evidence regarding the use of these apps.

The fact that most of the participants declared that they were using WhatsApp and Facebook Messenger corroborates descriptions in the literature of what the most popular mobile messenger apps within the field of medicine are, worldwide.^12^^,^^13^^,^^14^^,^^15^ Since the 1990s, these technologies have changed daily human activities, not only individually or economically, but also socially. A notable percentage of the participants stated that they used apps for medical issues frequently (daily, weekly or monthly), which demonstrates the applicability of such tools. As described by Giodano et al., these apps are an ideal tool for quick reference, as well as for clinical, academic and propaedeutic endorsements or for communication between healthcare professionals and patients, because of the inherent characteristics of mobile apps.^16^

The effect of using a secure messaging app (WhatsApp) for medical consultations in an emergency department was assessed in a randomized controlled study. Comparisons were made with consultation conducted by telephone. It was shown that use of the app (i.e. the intervention group) reduced the median length of stay in the emergency department (240 minutes versus 277 minutes) and reduced the median time spent on consultations (158 minutes versus 170 minutes).^17^

A British study assessed the implementation of the WhatsApp service within emergency surgical teams, in which the team members (n = 40) exclusively used WhatsApp for 19 weeks. It was demonstrated that use of this instant messaging tool promoted better communication of instructions, faster communication between interns and for attendance, and a flattened hierarchy among the team members.^18^

Another important study, conducted within the field of cardiovascular medicine, assessed the efficacy of WhatsApp for attending cases of ST-segment elevation myocardial infarction (n = 108) in rural areas in Turkey. It was observed that use of this app had a positive impact on triage and early activation of the cardiac catheterization laboratory, reduced door-to-balloon time and was an approach in keeping with international guidelines.

Thus, from these different reports, it can be seen that instant messaging apps are an efficient communication tool that enables resolution of problems within different medical specialties.

Regarding the legal aspects of use of IMS apps, our study showed that around 63% of the participants did not have any legal knowledge or had pursued non-literature-based knowledge in relation to the use of instant messaging apps for case discussions, imaging sharing and patient guidance.

In Brazil, even though more than three decades have gone by since the initial experiences with telehealth, legislative codification of telemedicine services into law, along with regulatory policies, remain at an early stage and information regarding this has not been disseminated among healthcare professionals. In contrast, in the United States and Germany, the use of telehealth services is underpinned by laws and regulations, thus resulting in bettering of medical practice and physician-patient relationships, given that the pre-specified policies avoid further prosecution and ethical concerns. Therefore, it is likely that thorough analysis of the legislature around the world is required in order to adapt and implement all the relevant medical jurisprudences for the scenario in one specific state in Brazil.

Medical practice is also constantly influenced by the approaches provided through instant messaging apps. The respondents in the present study perceived that the impact may be either positive or negative. General concerns relating to data protection and privacy are certainly relevant, and these ought to legitimate regulative intervention to avoid misuse and medico-ethical issues.

In agreement with previous studies, we observed that instant messaging services are an alternative way for physicians to communicate with patients or their families, and that these services present several advantages.^24^^,^^25^^,^^26^ The perceived benefits that have been observed within medical practice include reduction of medical errors^31^ due to rapid online consultation and information sharing; strengthening of physician-patient relationships, related to creation of “dedicated conversation channels”; democratization of medical management,^21^^,^^22^ since team-based decision-making can be implemented and patients’ preferences can also be considered; and most importantly, increased access to healthcare services, through reaching out to remote and socially vulnerable populations.^23^

In a Malaysian study, it was perceived that use of mobile messaging apps had a positive effect regarding coronary artery disease patients’ knowledge of and adherence to a healthy lifestyle. It was concluded that such tools, specifically WhatsApp, are useful additional mechanisms within current medical practice.^27^ Another previous randomized controlled trial assessing the use of instant messaging software for following up patients who were undergoing peritoneal dialysis showed that there was a higher degree of satisfaction among those using online approaches than among those in the traditional group.^28^

### Strengths and limitations of this study

This study raises awareness regarding the necessity for both legal and medical regulations for the use of instant messaging within medical practice. It is a pioneer in that it not only demonstrates the frequency and types of use of the main application (WhatsApp) but also demonstrates the use of other equivalent platforms.

The main limitation of the present study was in relation to the data collection process. Most of our participants were medical students, and this may have been a bias factor, given that when they were in contact with patients they would preferably be under the responsibility and supervision of a medical professor, to guide their clinical approach and management, which could have impacted on the participants’ behavior. Our research group tried to obtain support from the Brazilian national board of physicians to expand the survey nationwide among medical doctors, but this has not been possible so far.

Furthermore, the survey link was sent through an outsourcing procedure on the University website. This did not allow access to information on the exact number of people who received the email but did not even open it and the number who did open it. 

Also, the study was based on self-reported data, for which the rate of return of responses tends to be lower. It was impossible to assess how representative our sample was, in relation to the entire number of people who opened the email.

On the other hand, the use of a self-reported questionnaire may have been a strength, given that there was no interviewer creating bias through selection of answers and that the responders had autonomy.

## CONCLUSION

This study demonstrated how popular instant messaging apps have become among physicians and medical students nowadays, following the global trend. However, at the same time, it demonstrated how little both physicians and medical students know about the legal implications of the use of these tools. Therefore, it is advisable that regulatory legislation should be brought forward. Moreover, groundbreaking standard operating procedures should be proposed in order to ensure safety and security for all parties involved (physicians, patients, medical students and so forth). This is especially needed in developing countries such as Brazil, where regulations on this matter remain scarce.
